# Intelligent System/Equipment for Quality Deterioration Detection of Fresh Food: Recent Advances and Application

**DOI:** 10.3390/foods13111662

**Published:** 2024-05-25

**Authors:** Dianyuan Wang, Min Zhang, Qiyong Jiang, Arun S. Mujumdar

**Affiliations:** 1 State Key Laboratory of Food Science and Resources, Jiangnan University, Wuxi 214122, China; wdy292988976@163.com (D.W.); qiyongjiang@163.com (Q.J.); 2 Jiangsu Province International Joint Laboratory on Fresh Food Smart Processing and Quality Monitoring, Jiangnan University, Wuxi 214122, China; 3 China General Chamber of Commerce Key Laboratory on Fresh Food Processing & Preservation, Jiangnan University, Wuxi 214122, China; 4 Department of Bioresource Engineering, Macdonald Campus, McGill University, Ste. Anne decBellevue, QC H9X 3V9, Canada; arunmujumdar123@gmail.com

**Keywords:** fresh food, quality deterioration, intelligent detection equipment, supply chain

## Abstract

The quality of fresh foods tends to deteriorate rapidly during harvesting, storage, and transportation. Intelligent detection equipment is designed to monitor and ensure product quality in the supply chain, measure appropriate food quality parameters in real time, and thus minimize quality degradation and potential financial losses. Through various available tracking devices, consumers can obtain actionable information about fresh food products. This paper reviews the recent progress in intelligent detection equipment for sensing the quality deterioration of fresh foods, including computer vision equipment, electronic nose, smart colorimetric films, hyperspectral imaging (HSI), near-infrared spectroscopy (NIR), nuclear magnetic resonance (NMR), ultrasonic non-destructive testing, and intelligent tracing equipment. These devices offer the advantages of high speed, non-destructive operation, precision, and high sensitivity.

## 1. Introduction

Primary processed agricultural products such as freshly harvested fruits and vegetables, meat, and aquatic products, are conventionally referred to as fresh food. The fresh food market has grown globally especially over the past few years. Indeed, it has steadily grown to play a significant role in the overall economy. Big data indicates that in 2022, the commerce of fresh food in China reached CNY 560.14 billion. Fresh food purchases have been much more frequent in recent times, particularly during the COVID-19 pandemic [1]. However, fresh food is less processed and has the characteristics of high water content, perishability, seasonality, and short shelf life [2]. It is easily damaged in the processes from harvesting to consumption, causing various physiological and biochemical reactions in tissues and resulting in the decline of product quality and its nutritional value [3,4,5]. This causes significant economic losses to both consumers and suppliers. According to the available statistical data, the loss rate of fresh fruits and vegetables is as high as 20% in China every year, accounting for a large proportion of the total loss [6]. How to restrain the quality decline of fresh food is a difficult problem for the entirety of the supply system. The objective of this study is to identify and review available smart technologies that can ensure product quality. This can help reduce financial losses by utilizing real-time online detection devices for quality degradation of fresh food.

The research on fresh food quality deterioration detection tends to be more automated and intelligent with the advent of the information age and rapid developments in science and technology [7]. The development of equipment manufacture is supported by the Internet of Things, intelligent sensors, and cutting-edge algorithm systems. In the food sector, intelligent detection equipment is manufactured and used increasingly frequently. Intelligent detection equipment is based on intelligent detection technology, combined with high sensitivity sensors, computers, and equipment to transmit signals to the terminal display integrated system [8]. Compared with traditional equipment, intelligent equipment reduces labor costs and improves detection precision as well as efficiency. In recent years, the development of cost-effective, rapid, non-destructive, sensitive, and non-invasive intelligent testing equipment has attracted widespread attention [9], being used in meat freshness detection [10,11], fruit and vegetable quality classification [12,13], seafood quality monitoring [14,15], damage detection of grain [16], and food traceability [17].

In the process of postharvest storage and transportation of fresh food, some components of volatile and non-volatile substances are invariably lost. Some effects of quality deterioration often occur, such as shrinking, browning, soluble solid content reduction and undesirable odor generation [4,18]. Generally, high-quality products are selected through screening, classification, and some pretreatments. Following such processes, the production of some undesirable products can be averted, and thus, potential damage of product batches can be reduced. It also can maintain the freshness of products during storage and transportation. The use of intelligent detection equipment to detect the quality of fresh food can achieve hierarchical selection, determine the maturity and freshness of quality parameters, and predict its shelf life. In addition, it can also monitor product quality in real time and respond to the quality changes within the product at any time. At the same time, food traceability can be realized. Consumers can obtain relevant information on fresh products, realizing the identification of production location, harvest date, variety, authenticity, etc. Therefore, the quality of fresh food is ensured throughout the supply chain.

**Figure 1:**
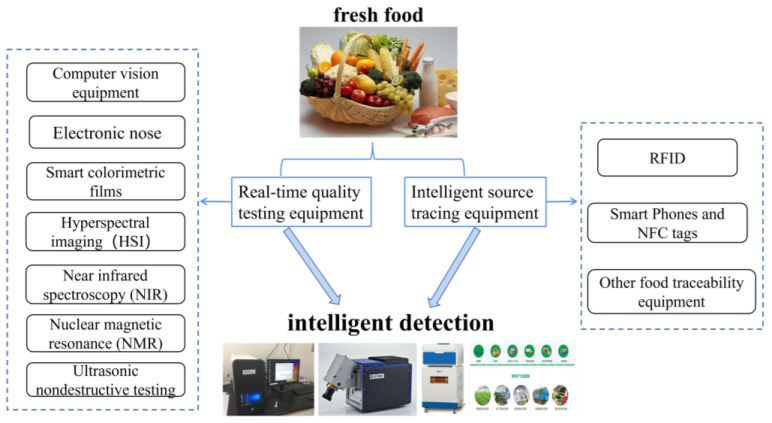
Summary of intelligent detection equipment for quality deterioration in fresh food.

Based on the techniques of sensory bionics, spectral imaging, nuclear magnetic resonance, and ultrasonic, this paper discusses several fast, non-destructive, highly sensitive intelligent testing equipment that can monitor the quality of fresh food online, and also presents an outline of the description of the traceability systems in use (Figure 1).

## 2. Intelligent Detection Equipment Based on Sensory Bionics

The operation of traditional detection equipment is complicated and time-consuming, the equipment cannot realize real-time online detection, and the price of the equipment is high. Therefore, it is necessary to develop intelligent real-time online detection equipment. Sensory bionic detection equipment aims to use biological sensors to imitate human vision, smell, touch, etc., obtaining product characteristics information. Then, the data are analyzed in combination with computer algorithms, and real-time online detection is thus realized. Computer vision equipment, intelligent packaging film, electronic nose, and other equipment are widely used in sensory bionic detection systems. Table 1 shows the application of intelligent detection equipment for monitoring fresh food.

### 2.1. Computer Vision Equipment

**Figure 2:**
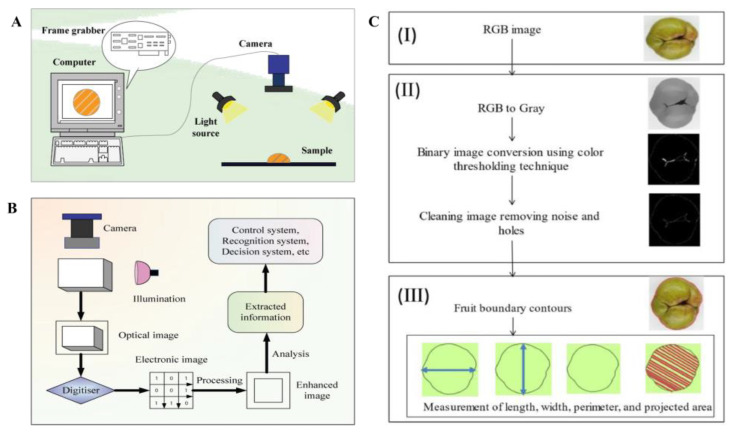
Schematic diagram of computer vision equipment. (**A**) The device is composed of intelligent camera, light source system, sample display area, and computer. (**B**) The working principle of computer vision equipment includes image acquisition, preprocessing, segmentation, and advanced image processing [50]. (**C**) Computer vision equipment measures steps as the physical characteristics of an apple [51].

Computer vision equipment uses intelligent sensors to simulate the human vision system. Through intelligent image capture equipment, the characteristic information of samples is collected and sent to the computer for image processing, and the target area is analyzed and identified [46]. Through it, the detection and classification of fresh food can be realized. Computer vision equipment is mainly divided into two parts: device for processing information and device for acquiring images [47]. The equipment is composed of image capture equipment, light source, image acquisition card, and computer, as shown in Figure 2B. The commonly used image capture equipment is the HD camera acquisition equipment (CCD camera) as a photo tool, responsible for taking photos of the sample under test and capturing the target image. The light source often used is LED lamp to achieve illumination stably. The task of the computer is to handle and analyze the image data by creating the right algorithms, including the features of color, texture, shape, and space [48,49]. The defect percentage can be calculated to judge the product quality to achieve the purpose of classification according to Formula (1). Dairath et al. [49] researched and developed a prototype robotic fruit picking cum grading system based computer vision using mango as an object. The specific steps of this computer algorithm include color scheme conversion, segmentation, background subtraction, morphological operations, bitwise operation, and percentage calculation. The results showed that the algorithm quickly realizes the quality detection and grading of mangoes in only 0.0125 s.

(1) $$\mathrm{Defect}\;\mathrm{percentage}\;(\%)=\frac{d}{f}\times 100$$

where *d* is the number of pixels of the defective product, and *f* is the number of pixels of fresh product. Those whose percentage values exceed a given threshold are classified as defective products.

More and more computer vision equipment has been created as a result of the advancement of computer vision technology. There are abundant applications for computer vision in the aspect of food testing, such as fruit and vegetable defect inspection and quality classification [52]. Zhu et al. [19] studied a new double-layer classifier based on computer vision, which could achieve banana grading, ripeness classification, and defect detection. The equipment was composed of two layers of classification system. The first layer classifier was SVM classifier. Through the setting algorithm, the banana immature, mature and over-mature population was separated and the accuracy reached 98.5%. The second classifier was the YOLOv_3_ system, which detected the defect area of ripe bananas. According to the size of the brown spot area, bananas were divided into medium and mature groups to complete the classification task of banana maturity, which solved the problem that small defects of bananas were difficult to detect. Su et al. [20] used a three-dimensional depth camera as an image acquisition device to acquire three-dimensional shape information of potatoes. After applying the depth algorithm, they effectively assessed the style features in the 3D model of potatoes and realized the categorization of potatoes based on quality by automatically detecting convex, curved shape, sag, and other appearance faults. In addition, the device also constructed a virtual potato 3D model, which can rotate 360 degrees and detect samples in multiple views to achieve the purpose of quality tracking. Benalia et al. [21] developed a FIG sorter to perform its real-time online grading. Because the object is moving and randomly oriented in real-time online detection, the image acquisition must be synchronized with the sample. Therefore, a fast image processing algorithm must be available. Differently from traditional cameras, a new image segmentation technique was developed in this study. They used two progressive color cameras (JAI CV-M77: Manufacturer: JAI Company; Denmark) to form a detection unit, providing RGB images (512 × 384 pixels). Then, the different images were segmented and processed to realize the grading.

Computer vision system has the advantages of fast and non-destructive operation, and high accuracy. Compared with traditional testing equipment, it greatly improves the efficiency of food testing. Using computer vision equipment can not only realize automatic classification and detection, but also be economical and hygienic. It can eliminate the interference of artificial subjective factors. However, there are also some difficulties in the practical application. As image acquisition equipment such as an intelligent camera is easily affected by environmental conditions (illumination, weather conditions, high humidity), image acquisition results will appear to have bright color differences, instability, and other phenomena [50], resulting in errors. Hence, future research on computer vision equipment will concentrate on enhancing precision and creating smarter, higher-resolution picture acquisition equipment. Although computer vision systems can simulate how the human eye captures color images, it is still challenging in areas such as early decay, minor bruising, and other invisible defects. Zhang et al. [53] present a detailed solution showed that it was possible to combine computer vision with other new imaging techniques, 3D technologies, etc., to develop more advanced intelligent equipment. The selection of good characteristics for classification is an important process of feature extraction [54]. Xu et al. [48] pointed out that computer vision was a simple simulation or imitation of human vision and could not adapt to changes in food types as flexibly as the human eye. A similar point was suggested by Kaushal et al. [55], saying that the future direction of computer vision was to improve the algorithms for food recognition to be close to human vision and easily able to deal with the variability between different food products. Therefore, it is important for researchers to increase the degree of simulation of computer vision to make it behave more like the human visual system to improve detection accuracy.

### 2.2. Intelligent Packaging Film

A modern variety of visual packaging equipment known as intelligent packaging film can instantly transmit real-time status information of foods [56]. It is often used in the preservation and quality testing of fresh food to reflect the deterioration of food in transportation and storage. The packaging films are usually composed of solid substrates (polysaccharides) and pH indicators (natural pigments such as anthocyanins, carotenoids, betaine, etc.) [57,58]. With the extension of storage time, the pH value of the internal ingredients of fresh food will change. It causes a color change in the film to convey the deterioration information of the product. Due to the progress of sensor technology, many studies have been conducted to embed low-cost sensors into films to improve the detection efficiency of intelligent packaging films. Sensors used in the device include colorimetric sensors, temperature and time sensors (TTIs), biosensors, and humidity sensors [59]. In addition, a good packaging film should have excellent mechanical properties, low water vapor permeability, good UV resistance, and strong antibacterial [60,61,62]. Tensile strength (TS) and elongation at break (EB) reflect the flexibility and mechanical resistance of food packaging film, respectively. Water vapor transmittance (WVP) is very important for the moisture resistance of the film [57]. The specific calculation formula is as follows (2)–(6):

(2) $$\mathrm{Tensile}\;\mathrm{strength}\;(\mathrm{TS})=\frac{F_{max}}{A}$$

(3) $$\mathrm{Elongation}\;\mathrm{at}\;\mathrm{break}\;(\mathrm{EAB})=\frac{L-L_{0}}{L_{0}}$$

where *F_max_* refers to the maximum force of the film before fracture, *A* is the cross-sectional area of the film, *L* is the elongation of the film before fracture, and *L*_0_ is the initial length of the film.

(4) $$\mathrm{WVP}=\frac{W\times x}{t\times A\times \Delta P}$$

where *W* is the weight increased during the test, *x* is the average thickness of the film, *t* is the time of weight growth, *A* is the penetration area of the film, and Δ*P* is related to temperature.

(5) $$\Delta E=\sqrt[{2}]{{\left({L_{0}}^{*}-L^{*}\right)}^{2}+{\left(a^{*}-a\right)}^{2}+{\left(b^{*}-b\right)}^{2}}$$

(6) $$\mathrm{WI}=100-\sqrt[{2}]{{\left(100-L^{*}\right)}^{2}+a^{2}+b^{2}}$$

where *L*_0_*, a*, and b* represent the color parameters of the standard whiteboard, and L*, *a*, and *b* represent the color parameters of the film sample. In addition, L* is brightness, a* is red/green, and b* is yellow/blue.

Hashim et al. [22] investigated a novel pH colorimetric film to check the freshness of shrimp. It was made of sugarcane wax, butterfly pea flower extract, and AGAR as the substrate. The results demonstrated that the intelligent packaging film could accurately identify shrimp quality and monitor shrimp quality changes. Liu et al. [23] studied a new intelligent colorimetric film for the real-time detection of mushroom freshness changes. The film used nanotechnology to enclose blueberry anthocyanins into protein–polysaccharide complexes, which were then added to the membrane matrix. This method not only improved the stability of anthocyanins, but also improved the properties of the films. With the increase in the number of storage days, the intelligent colorimetry film changed from purple to red, indicating that it could well represent the quality changes in mushrooms. A TTI is typically used as a label on film packaging [63]. The product will exhibit color change and mechanical deformation with accumulation of time and temperature. Food temperature history can be tracked in real time, and product deterioration can be analyzed [64]. Xu et al. [24] studied a simple TTI device and attached it to food packaging film as a label. The device judged the quality of sashimi according to the reaction of tyrosinase and tyrosine. The results revealed that the TTI altered with time and temperature, changing from colorless to dark, indicating a decline in the quality of the turbot sashimi. Popa et al. [65] developed a low-cost commercial intelligent detection device which consisted of gas, temperature, and humidity sensors and a computer. Taking an onion as the test object, it was shown that the device could detect the basic information of onion slices in vacuum packaging, and it could observe the change in product quality with time. Therefore, the sensor system can be used in smart packaging film. And it can combine with smart phones to further monitor the storage status of fresh food.

Intelligent packaging film is inexpensive, quick to apply and remove, and easy to observe. It has a broad range of applications, particularly for cold chain logistics transit and storage. However, fruits and vegetables are currently employed far less frequently than meat and seafood when it comes to intelligent packaging film. According to a review by Bhargava et al. [66], most of the packaging films had unsatisfactory tensile strength and elongation at break, resulting in low mechanical properties. The film with poor antimicrobial resistance is susceptible to degradation when it is contaminated with bacteria [67]. Natural pigments such as anthocyanin are often added to packaging films due to their antimicrobial properties. However, it is extremely unstable [57]. It is easily affected by external factors and loses its original color characteristics, leading to a decrease in detection accuracy [68]. Therefore, it is necessary to improve the mechanical properties, antibacterial strength and reliability of the membrane through appropriate technology and materials. More importantly, the development of lower cost sensors to embed smart packaging film is also the direction of future research. This will make the intelligent packaging film in the field of fresh quality detection more widely used.

### 2.3. Electronic Nose

**Figure 3:**
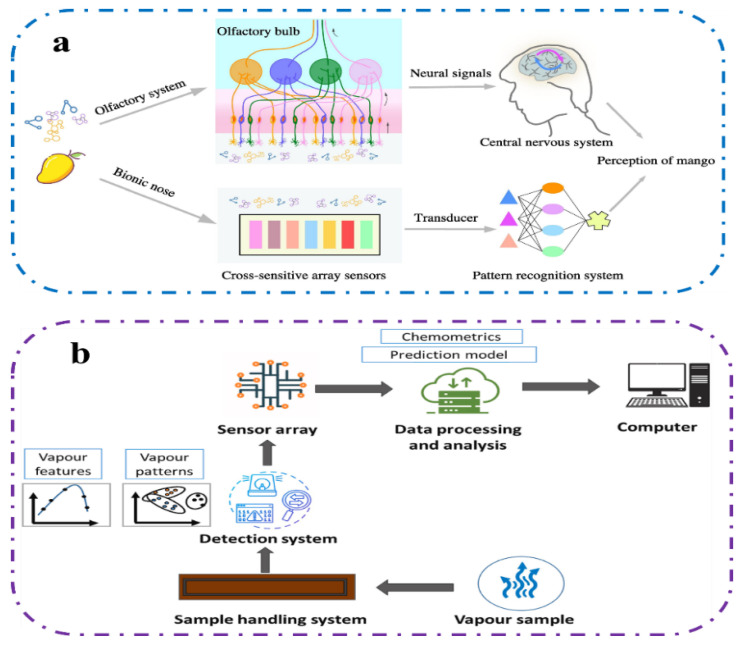
Schematic diagram of electronic nose device. (**a**) A comparison between electronic nose and human olfactory processes [73]. (**b**) The composition and working principle of electronic nose system [74].

Using a variety of sensors and pattern recognition technology, an electronic nose simulates the biological sense of smell and can detect and recognize complex aromas [69]. The electronic nose consists of gas sensor, signal collector, and computer system [70]. Figure 3 shows how an electronic nose works and its working principle is very similar to the human olfactory recognition process. When the volatile molecules in the sample under test come into contact with the sensing material of the gas sensor, it will immediately convert the response into an electrical signal. Then, through pattern recognition, the sensor detects and characterizes these changes for classification, thus obtaining qualitative or quantitative analysis [71,72]. The main multivariate classification methods used in an electronic nose are principal component analysis (PCA), linear discriminant analysis (LDA), and partial least squares discriminant analysis (PLS-DA). The data model is built based on the algorithm. And the performance of the classification model is evaluated by sensitivity, specificity, accuracy, and error rate [27], which can be can be calculated by Formulas (7)–(10):

(7) $$\mathrm{Sensitivity}\;(\%)=\frac{TP}{\left(TP+FN\right)}\times 100$$

(8) $$\mathrm{Specificity}\;(\%)=\frac{TN}{\left(TN+FP\right)}\times 100$$

(9) $$\mathrm{Accuracy}\;(\%)=\frac{\left(TP+TN\right)}{TOTAL}\times 100$$

(10) $$\mathrm{Error}\;\mathrm{rate}\;(\%)=\frac{\left(FP+FN\right)}{TOTAL}\times 100$$

where *TP* is true positive, *TN* is true negative, *FP* is a false positive, and *FN* is false negative.

In recent years, Shi et al. [75] reviewed the uses of an electronic nose for monitoring fresh food, indicating that the electronic nose was crucial for the growth and development of the food industry. Ezhilan et al. [25] designed a low-cost portable electronic nose designed specifically to evaluate the freshness of broccoli. A sensor array composed of six chemo-resistive sensors capable of detecting various volatile organic compounds was placed in the top space of the test sample. PCA, centroid analysis, and whole-chain cluster analysis were used for quality discrimination. Grassi et al. [26] developed a low-cost electronic nose device that can detect different marine products. It consisted of four specific metal oxide sensors, a photoionization detector, and a battery. According to the study, the electronic nose device used special algorithms and classification models to detect the freshness of mullet, red mullet, and cuttlefish and to classify different seafood products. In the future, this device can be applied to seafood management in the market. Haghbin et al. [76] developed and designed a low-cost and efficient detection system using an electronic nose and machine learning technology. It can realize the online monitoring and classification prediction of gray mold in kiwi fruit during postharvest storage. Wijaya et al. [28] developed a portable electronic nose for proctoring and testing beef freshness. The device was developed with low-cost hardware and equipped with a Molen system, which could be combined with smart refrigerators and smart phones to conduct real-time monitoring and analysis during distribution and storage. And it finally released beef quality information to the end users. The equipment was perfect for distinguishing between fresh and spoiled beef.

The electronic nose has the characteristics of high accuracy and fast, simple, and non-destructive operation, and can provide online analysis, which has attracted wide attention in the food field. However, the detection results of an electronic nose are susceptible to the influence of sensor characteristics (sensor time drift, redundancy) and ambient atmosphere [77,78]. In recent times, most electronic nose applied in the laboratory have failed to achieve large-scale application. In addition, electronic nose measurement needs to be performed by using many complex algorithms. Therefore, as science and technology continue to develop, more sophisticated sensor performance systems and simpler algorithms will be investigated and produced, and the use of electronic noses for food detection will become increasingly widespread.

### 2.4. Other Detection Devices

As an efficient and intelligent technology, intelligent sensing technology has made great achievements in practical applications (including medicine, agriculture, food, and other fields). There are some new examples of detection equipment based on other sensory bionic systems.

ABS Laboratories in the United States has developed a smart portable device—Food Sniffer. This equipment is a new type of fast detection equipment, which is specially used to evaluate the freshness of meat. Castrica et al. [79] evaluated the freshness of cod fillets with a food sniffer. The device consisted of a metal oxide sensor, a message processor, and a Bluetooth device. In order to obtain product information right away for the user, the gas sensor measured the concentration of volatile compounds, turned it into an electrical signal, and sent it to the smartphone using a specialized algorithm. The results demonstrated that the apparatus accurately informed customers about the condition of cod fillets in real time and showed promise as a tool for identifying food quality decline. Zhang et al. [80] studied a smart sensing manipulator system (FSIMS) that mimicked the human sense of touch to automatically grade avocados according to their ripeness. The system included a mechanical clamp, a flexible sensor, and a monitoring part. The working principle was to clamp the avocado sample under test, and then the flexible pressure sensor sensed the external force to determine the different pressure values at the contact site. And it could identify the hardness and grade the maturity of the fruit. The results showed that the recognition rate of platform was 97.5%. Chen et al. [81] designed an artificial olfactory sensor using seven flexible metal–organic frames (MOFs) that could highly and selectively detect volatile organic compounds to determine the health of tomatoes. The equipment could detect low reactive volatile organic compounds that were not sensitive to traditional dyes. Under different humidity and time conditions, the detection results were reproducible and stable, and the tomato was evaluated as to whether it was spoiled. The usage of auditory sensors is growing in a variety of sectors, including laboratory analysis, pollution monitoring, food analysis, and psychiatric condition [82]. It mainly uses the change in sound waves to reflect the quality information inside the food. Through computer analysis of these sound signals, the crispness and maturity of fruits and vegetables and other fresh foods are evaluated to achieve online control of fresh products. Foerster et al. [83] used acoustic sensors to perform computerized resonance analysis, conduct internal quality inspection of asparagus, and check the cavitation phenomenon of asparagus to achieve excellent quality automatic classification.

## 3. Spectrum and Imaging Testing Equipment

Spectral and imaging equipment is used to detect the quality of food by means of image processing, spectral analysis, imaging technology, computer technology, and chemical measurement. Generally, it can realize online detection through the difference in atlas signal between samples, which will not cause damage to the product, and can solve the problems of time-consuming operation and low accuracy of traditional equipment. And the device has the advantages of simple and fast operation and the real-time detection of internal composition changes. By analyzing these changes, food quality information can be obtained and the quality of fresh food can be judged. This review mainly introduces near-infrared spectroscopy equipment and hyperspectral imaging equipment.

### 3.1. Near-Infrared Spectrum Equipment

**Figure 4:**
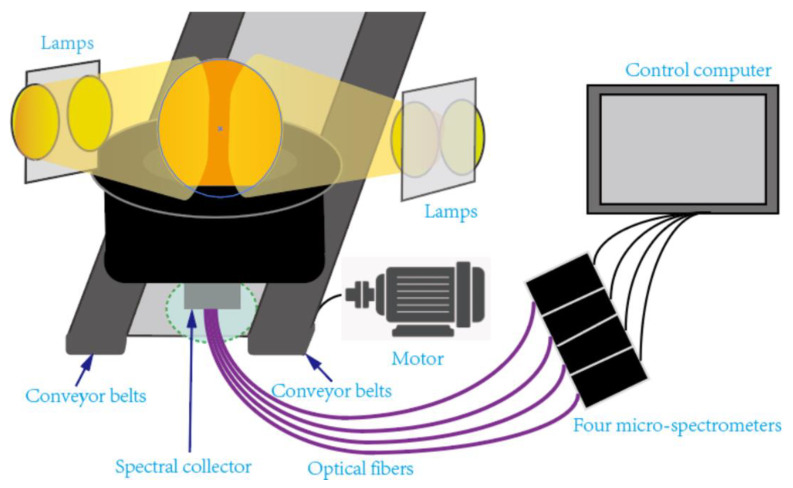
Schematic diagram of online near-infrared spectrum measurement system [85].

The wavelength of near-infrared spectroscopy (NIR) is between 780 and 2500 nm. Because of the vibrational properties of molecules, organic compounds will transition from the ground state to higher energy levels. These transition leads to energy absorption (or emission), and the change in absorbed (or transmitted) energy with wavelength is represented by a spectrum. Each bond of the molecule has a unique vibration pattern and frequency. When the sample is analyzed with near-infrared spectroscopy equipment, the chemical bonds receive energy that matches its vibration frequency, and the light energy will be absorbed [84]. The chemical composition of the product is represented by the near-infrared spectrum, which varies based on the composition of the sample. A typical online near-infrared spectroscopy measurement system is shown in Figure 4. The system was composed of a light source, a transmitter, a micro spectrometer, a special optical fiber, a spectral collector, and a computer. Firstly, the measured sample was placed on a tray and transported at a certain speed to the spectral collector using a conveyor belt, which triggered the spectral measurement. The light emitted by the light source penetrated the fruit under test and was transmitted to the spectrometer through optical fibers. Then, the spectral data were collected and stored on a computer [85]. The NIR equipment has the advantages of fast and non-destructive operation and online real-time detection, and can achieve quality tracking from harvest to postharvest storage [86]. And it is widely used in the quality assessment of fresh food such as beef, mangos, and peaches [87,88,89].

Choi et al. [29] designed a portable non-destructive detector focused on VIS/NIR reflectance spectroscopy for Asian pears. The device was a handheld device with a portable battery, built-in sensors, and a result display. Data stored inside the device were transmitted via Bluetooth to the smartphone or computer of users. The results showed that the instrument could easily and quickly determine the sugar content of pear, and establish a reliable mathematical model. The device could forecast the sugar levels of any kind of pears and was repeatable at various temperatures. De Marchi et al. [30] used near-infrared spectroscopy to predict beef quality traits online, and measured them directly on the carcass surface with optical fiber probes to establish a near-infrared spectral data model to evaluate beef pH value, color index, and shear force. The results indicated that this system may be an effective tool for online beef quality prediction. Xu et al. [31] developed an online near-infrared detection method depending on double-cone roller transportation to ascertain the amount of soluble solids (SSCs) in apples. This system consisted of an NIR detection part, transportation part, classification operation part, and computational control part. The samples were measured at specific locations and regions. Through spectral measurement and stoichiometric data analysis, the optimal prediction model of SSC was obtained, and a more accurate classification measurement of apple was realized. Xu et al. [32] evaluated the semi-transparency of pineapple pulp using a vision-near-infrared spectroscopy (VIS/NIR) platform developed in the laboratory. The device measured three batches of pineapple slices with different delivery dates, which consisted of nine halogen lamps and two spectral sensors. According to spectral information, partial least squares regression analysis (PLS) and probabilistic neural network (PNN) model, the samples were divided into three categories: non-translucency, slight translucency, and heavy translucency. This study showed that VIS/NIR could quickly and non-destructively assess the internal quality of pineapples and had the potential to apply non-destructive quality assessment to other large fruits.

With the advantages of being quick and inexpensive, near-infrared spectroscopy may achieve online detection and determine a range of components simultaneously. However, spectral measurement is easily affected by environmental conditions, resulting in errors in the accuracy and precision of measurement results. In addition, the stability of instruments and equipment is poor, and the analysis method of mathematical model is complex, hence not being widely applied to enterprises and factories. To enhance the ability of data analysis and achieve accurate product classification, a more precise and straightforward algorithm model must be established.

### 3.2. Hyperspectral Imaging Equipment

**Figure 5:**
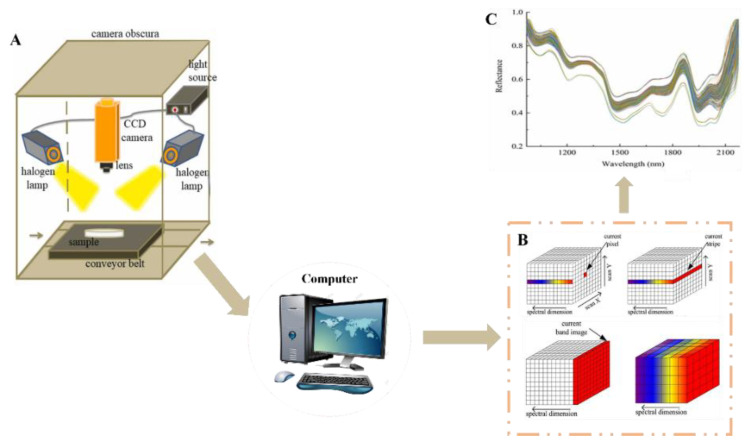
Hyperspectral imaging equipment structure diagram. (**A**) The system diagram of HSI. (**B**) Hyperspectral image 3D data matrix. (**C**) Hyperspectral reflectance diagram [97].The colors in (**B**) represent the different spectral channels in the hyperspectral image (bands between 969–2174 nm). Different colors correspond to different bands. The colors in (**C**) represent samples, and different colors correspond to different samples.

Hyperspectral imaging (HSI) is an innovative method of intelligent detection. To obtain three-dimensional spectrum data and continuous narrow-band image data with high spectral resolution and spatial precision, it integrates imaging and spectral technology. This is accomplished by detecting the essential features and data of the tested sample [90]. Hyperspectral imaging is progressively being used to evaluate the quality of fruits and vegetables, seafood, cereals, and other commodities due to its benefits in detecting ability, precision, and spectral information [91,92,93,94,95]. In addition, it can also be used to detect the content of moisture, fat and protein in food [96]. A schematic diagram of hyperspectral imaging equipment is shown in Figure 5; it mainly includes a spectral scanner, high-performance CCD camera, light source, data collector, image processor, and so on. The instrument is integrated in a cassette that is not affected by ambient light. However, the raw data image is often disturbed by a variety of noises when acquiring the image. These noises not only affect the visual effect of the image, but also reduce the application accuracy. In order to reduce noise and error caused by light sources, hyperspectral images need to be corrected. Images are usually represented mathematically as matrices. As shown in Formula (11) [97], the corrected image is obtained after subtraction and inverse matrix operations.

(11) $$R=\frac{I-D}{W-D}$$

where *R* is the corrected image, *I* is the original spectral image, *W* is the whiteboard data, and *D* is the blackboard data. They are represented in matrix form.

Huang et al. [35] proposed a hyperspectral imaging system composed of hyperspectral imager, halogen lamp, sample tray, and computer to obtain hyperspectral images of persimmon, analyze spectral data to establish an optimal bruise detection model, and identify persimmon health/injury. The results showed that the classification accuracy could reach 100%. At the same time, using the method of minimum noise analysis, the image segmentation of persimmon bruising area was carried out to accurately identify the damaged part of persimmon. This indicated that the hyperspectral system could realize the online detection of early damage of persimmon and could be used as an effective detection equipment. Qin et al. [33] developed a hyperspectral imaging system module that could monitor citrus canker in real time. The device consisted of twenty halogen tungsten spotlights as light sources. A spectroscope, two different wavelength bandpass filters, and a monochrome camera are used to obtain real-time images. The results showed that the classification accuracy of this device was more than 95%, and it could effectively detect citrus canker in real time. Tang et al. [34] combined hyperspectral information with image texture information to analyze the quality characteristics of a large number of pork samples. This work used SpecimIQ hyperspectral cameras (Specim, Spectral Imaging Ltd.; Finland) in a 400–1000 nm wavelength range to extract the image. An artificial neural network (ANN) was applied to establish a regression model to forecast the flesh quality and classification. The results indicated that adding texture analysis to hyperspectral imaging technology could significantly improve the accuracy of detection. Shao et al. [36] used hyperspectral imager, light source, and computer and other major equipment to classify sweet potato through hyperspectral imaging technology. In this work, a variety of algorithms were used to establish the classification model. The results showed that the SPA-LDA model had the best classification performance and the accuracy rate could reach 99.52%. It was proved that the equipment could accurately detect defects and provided a complete classification of sweet potato.

A large number of studies suggest that hyperspectral imaging can realize the rapid non-destructive online detection of fresh food quality. However, the hyper spectrum has certain defects, such as the complexity of hyperspectral imaging. Due to the large image size, the camera requires significant data storage and processing capabilities. Therefore, it is necessary to develop a hyperspectral camera with high resolution and high processing capabilities. Meanwhile, the amount of spectral information collected by this technology is large, leading to a large consumption of processing time, so it is necessary to improve the data processing technology, establish an effective model to refine the algorithm, and reduce the data processing time. In addition, hyperspectral imaging is prone to the impact of noise, lighting, and other environments due to dark current and uneven lighting. How to eliminate the impact of noise more effectively is a problem that future researchers need to pay attention to.

## 4. Nuclear Magnetic Resonance (NMR) Detection Equipment

Nuclear magnetic resonance (NMR) is a method to study the structure and properties of matter by using the magnetic properties of atomic nuclei and the effect of external magnetic fields. Its basic principle is that in a constant magnetic field, the nucleus with a non-zero nuclear magnetic moment spins around the applied magnetic field, which is called precession. Precession has a certain frequency, which is proportional to the strength of the applied magnetic field. On this basis, a fixed-frequency electromagnetic wave is added, and the intensity of the external magnetic field is adjusted, so that the precession frequency is the same as the electromagnetic wave frequency to produce resonance. At this time, the nucleus absorbs the energy of the electromagnetic wave and transitions from the lower energy level to the higher energy level [98,99]. Nuclear magnetic resonance is widely used in the field of food. It employs radio-frequency low-energy electromagnetic waves, which are not significantly absorbed by biological tissues. It can be used to make internal, non-destructive examinations of fresh food to determine their quality [100]. NMR can be used to assess the composition and structure of food samples, and to quantitatively determine the water, fat, carbohydrate, and protein content of food [101]. It can online detect the dynamic change in food during storage [102,103] and improve the quality of food while it is drying [104], freezing [105], and so on. Nuclear magnetic resonance is basically divided into high-resolution nuclear magnetic resonance (HR-NMR) and low-field nuclear magnetic resonance (LF-NMR) [106]. HR-NMR is not suitable for large-scale applications because it requires expensive instruments, high cost, and highly professional operators. Another technology known as magnetic resonance imaging (MRI) can view information about the internal organization of food [107]. In recent years, LF-NMR and MRI have been widely used in the non-destructive testing of fresh food due to their high sensitivity and rapidness [108].

Nuclear magnetic resonance equipment generally consists of a magnet, a pulse generator that generates a radio-frequency magnetic field, a signal detection receiver, a transmitter and/or receive coil, and a computer [109]. NMR is the most complex investigation method and one can employ spectroscopy, relaxometry, diffusometry, imaging, etc., or a combination of the above in low or high static magnetic field in one or more dimensions. Lv et al. [37] developed an NMR/MRI device combined with microwave vacuum drying that was capable of online measurement of moisture content and status. The device could swiftly assess the moisture change in corn kernels during microwave drying, which not only determined the drying end point directly from MRI, but also solved the problem of uneven drying by traditional microwave [110]. Nakajima et al. [38,39] built a low-cost lightweight handheld sensor made of a unilateral magnetic circuit and a planar radio-frequency coil. The sensor was a component of the magnetic resonance (MR) scanning system. It used the NMR relaxation method to quantitatively identify intramuscular fat in fresh beef, the proportion of fat and lean meat in tuna. The study demonstrated the lightweight handheld MRI tomograph was a kind of intelligent food detection equipment with great potential. Cheng et al. [40] monitored water dynamics during shrimp drying using LF-NMR and MRI. In this study, an NMR analyzer was used to measure transverse relaxation. And combined with imaging software to obtain magnetic resonance imaging, the authors evaluated the water state of shrimp during drying and observed the internal structural information of shrimp. Water content, texture profile analysis, and color change were used to evaluate the dryness and quality of shrimps. The results demonstrated that the technique was a quick and non-destructive way to guarantee the quality of seafood. Fu et al. [41] developed a novel non-destructive testing system based on low-field nuclear magnetic resonance (NMR) and deep learning neural network (DLNN) algorithms. Based on LF-NMR and MRI, the system characterized the water boundary state and distribution in dried longan fruit. Combined with the algorithm, the defective dried longan fruit was quickly and effectively predicted, and the healthy fruit was distinguished. 

NMR has the advantages of rapid, non-destructive, non-invasive, and direct determination of the internal composition information of samples. It is an important technology for online real-time detection and analysis of food quality deterioration, and has made great progress in many related fields. However, MRI equipment is currently confined to laboratory research. One of the main limitations is that benchtop MRI machines are expensive, costly, and require the examiner to have a knowledge base which is complex compared to other equipment. Consequently, the focus of future research should be on producing low-cost, user-friendly equipment as well as comprehensively increasing the hardware capabilities of NMR devices. Additionally, this equipment can be used in conjunction with other technologies to boost the stability and accuracy of detection.

## 5. Ultrasonic Testing Equipment

Ultrasonic waves are mechanical waves beyond the range of human hearing. The food industry has recently paid attention to the rapid development of ultrasonic technology. As a new and non-invasive online acoustic detection technology, ultrasound has the characteristics of non-destructive, rapid, and simple, and offers strong reliability [111]. It has been reported that changes in acoustic properties may be related to changes in the density of components in food [112]. Therefore, ultrasound can be used to detect changes in the property (density) of food tissues as a result of biochemical reactions. Recently, ultrasonic testing has been applied in the non-destructive testing of fruits and vegetables [113,114], drying [115], sterilization [116], storage, and preservation [117]. According to the literature, in non-destructive food testing, high-frequency ultrasound (low-power ultrasound) is frequently utilized to offer physical and chemical information about food [118]. The basic principle of ultrasonic non-destructive testing is to absorb and scatter waves when interacting with substances. By determining the changes in ultrasonic propagation speed (Formula (12)) and signal attenuation (Formula (13)), the correlation between them and the influencing factors of fruit quality can be judged, so as to evaluate product quality. This principle has been proven in the detection of fruit acidity, viscosity, and sugar content [119].

(12) $$v=\frac{d}{t}$$

where *d* is the thickness of the sample, and *t* is the flight time.

(13) $$\alpha =\frac{1}{d}\ln\left(\frac{A_{0}}{A}\right)$$

where *A*_0_ and *A* are the peak ultrasonic signal amplitude (V) at the beginning and end of the ultrasonic propagation path, respectively.

A review by Mizrach et al. [120] showed that various devices based on ultrasound have been created to supervise the physiological quality changes in fresh food such as fruits and vegetables during postharvest, storage, and transportation. And it can detect the texture and storage stability of fresh food. Hardness is one of the indicators to determine the maturity and storage time of fresh fruit. Feng et al. [121] realized the preservation of cucumbers after harvest by using ultrasonic treatment combined with controlled atmosphere storage. The results show that ultrasonic treatment can not only guarantee the quality of cucumber during storage, but also preserve the unique flavor of cucumber to the greatest extent. Kim et al. [42] developed an online non-destructive testing system based on ultrasonic technology consisting of an ultrasonic pulse generator, dedicated ultrasonic transmitting and receiving transducers, digital oscilloscopes, and computers to perform ultrasonic measurements and hardness tests on fresh apple samples. In this study, the apparent elastic modulus and breaking point of apple were measured by compression test, and the correlation between ultrasonic velocity, attenuation and apple hardness was analyzed. The results showed that the use of ultrasonic measurement was a reliable method to predict the hardness of apples, as well as evaluate their maturity and estimate their shelf life. Mizrach et al. [43] applied an ultrasonic non-destructive device to the whole avocado to measure internal changes during the softening process and assess its quality. The device used ultrasonic probe at different distances on the peel of the wave amplitude and traveled time to measure ultrasonic attenuation and speed. The hardness, dry weight, and oil content of fruit were measured, and the correlation between them was analyzed by establishing a specific model. The results indicated that changes in ultrasonic parameters during predicted storage could be used to assess hardness and oil content. It could be used to forecast when avocado fruit will ripen and how long they will last. Soltani Firouz et al. [44] designed an online non-destructive testing system to measure the ultrasonic characteristics of oranges through transmission mode to determine the degree of freeze damage. The system was combined with the SVM method to realize the online classification of healthy oranges and frozen oranges, and the accuracy reached 100%, thus ensuring the quality of oranges in storage, transportation, and distribution. Prado et al. [45] used an ultrasonic device to online non-destructively detect the fat content of raw ham and graded it into three groups. The researchers determined the ultrasonic velocity by measuring the ultrasonic travel time and sample thickness. Then, a data classification model was established based on ultrasonic velocity, and the accuracy of the final classification reached 88.5%. The results showed that the average ultrasonic speed was related to the fat content of ham, and the ultrasonic system was feasible to detect the quality of meat.

Ultrasonic testing equipment is relatively cheap, simple and energy saving, with high precision, non-destructive operation, and timely characteristics, has a very large market promotion value. However, there are limitations to ultrasonic testing equipment. For example, each fruit or vegetable has its specific ultrasonic response, traditional ultrasonic devices cannot be uniformly applied, the environment can easily have an impact on ultrasonic equipment, etc.

## 6. Intelligent Source Tracing Equipment

In the food supply chain, an efficient food traceability system (FTS) must be established. It may give customers complete product information, assure the quality and safety of food, and defend their fundamental concerns and rights [122]. Food traceability is the capacity to monitor and record the movement of food during the processing and distribution stages, enabling the traceability of the history or origin of the product at any moment [123,124]. In recent times, numerous traceability tools and systems have been created and used, involving fruit and vegetable processing [125], seafood transportation [126], animal food authenticity prediction [127], and water quality detection [128]. Researchers have developed more and more intelligent identification and traceability equipment based on advanced technologies such as sensor technology, big data, smartphones, and blockchain technology [129]. Table 2 summarizes the application of common traceability equipment for monitoring fresh food.

### 6.1. Radio-Frequency Identification (RFID) Equipment

**Figure 6:**
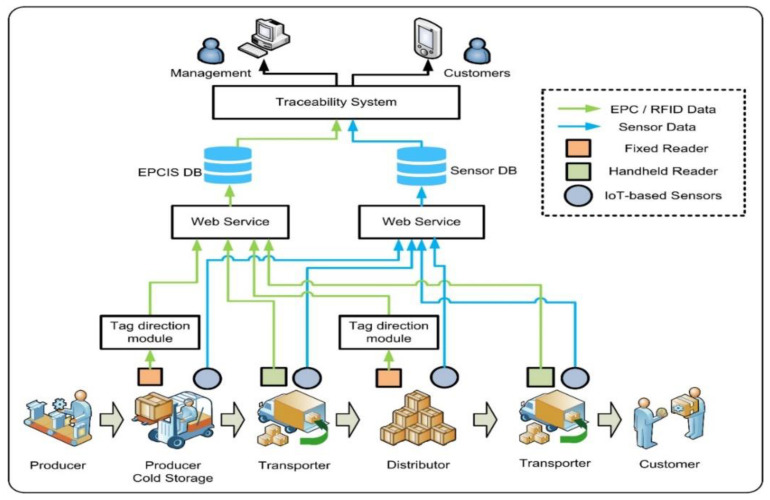
Food traceability system based on RFID technology. It uses RFID and sensors to collect temperature and humidity information to enable food traceability from production to consumer [141].

RFID tags can automatically identify and track the objects they are attached to. The information is stored in the memory of the tag, processed by the RFID reader, and sent to the back-end database for remote access and item parameter monitoring [141]. Also, it is able to track the whole food supply chain as shown in Figure 6, which is vital for ensuring the quality of fresh food. Zuo et al. [142] indicates that RFID is widely used in different aspects, such as food freshness detection, origin traceability and authenticity detection, product tracking, and cold chain monitoring, as well as the traceability of fresh agricultural products.

Fan et al. [130] developed a low-cost barcode–RFID bidirectional conversion device that improved traceability by preserving identity connections and data correspondence. RFID and barcodes could be transformed in both directions. A haptic industrial controller, an RFID reader module, an integrated printing module, and a barcode scanning module were all included in the device. The technique had been used for packing wheat flour and segmenting meat. The results demonstrated that the technology had been successfully used to track food. Using Internet of Things and RFID technologies, Urbano et al. [131] designed a traceability device composed of an RFID subsystem and information processing subsystem. RFID subsystems include RFID chips, microcontrollers, specific sensors, RFID readers, and necessary electronic devices. Orange and pumpkin temperature and cold chain monitoring demonstrate the system could recreate the cold chain and determine temperature conditions. This study addressed the issue of cost and technological interconnection in the traceability system by merging with Internet of Things technologies, which could more precisely implement the tracking and tracing of food conditions in the transportation process and ensure the quality of products. Abad et al. [132] designed an RFID smart tag for intercontinental cold chain logistics of fresh fish. The system consisted of intelligent tags and commercial readers. Smart tags included sensors, microcontrollers, memory chips and antennas for RFID communication. Commercial readers were available at any point in the supply chain to read and capture product data in real time. The results suggested that the RFID-based system offered the benefits of a large memory capacity, no personnel costs, the capacity to simultaneously read many labels, strong environmental endurance, and the ability to achieve real-time traceability and cold chain monitoring. Feng et al. [143] designed and tested a beef traceability system that integrated the RFID method with PDAs and barcode printers. This work proposed a conceptual model for information exchange and transmission based on data acquisition and traceability. The results indicated that the traceability system could efficiently follow the full cattle supply chain and offer real-time and accurate information about beef products. An intelligent traceability system combined with RFID technology and a molecular analysis algorithm (DNA) was evaluated by Cappai et al. [133], showing that the system was capable of providing traceability performance and may be utilized to safeguard the origin and brand of pigs as well as to monitor disease.

RFID is a technology that is incredibly promising and growing quickly. Compared with traditional tags and barcodes, it has advantages of high efficiency and rich and accurate information. The development of an intelligent sensor RFID tag and its application in the traceability system can not only meet the needs of consumers for food safety information, but also reduce food waste. However, there are also some challenges related to this technology, such as the cost and recovery of labels, limited scope of information reading, etc. Therefore, researchers should think about how to improve the algorithm, broaden the area of information gathering, decrease cost recovery in the future, and encourage the use of RFID in the food industry.

### 6.2. Smart Phones and Near-Field Communication (NFC) Tags

**Figure 7:**
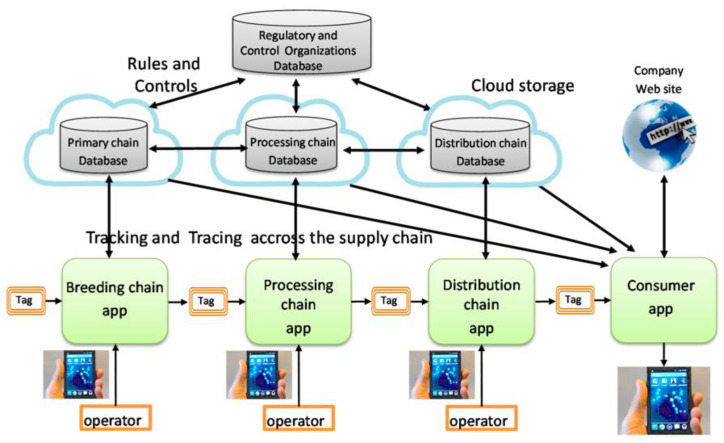
Architecture of a food traceability system based on smart phone and NFC. It is used to track and trace the entire supply chain [147].

In recent years, smart phones have developed rapidly and have become an indispensable part of human life. They have played an important role in food industry applications [144]. Smart phones are used to detect food quality, track food information, and balance dietary nutrition [145]. Smartphones have a number of advantages such as being light and portable, having strong mobility, being able to perform powerful calculations, and offering real-time monitoring. Smartphones are already being used as an effective tool to help consumers and managers track the origin and date of food products and gather information relating to quality parameters. Figure 7 shows the traceability detection of smart phones in the food supply chain. It mainly obtains the information consumers want by establishing data sets, scanning a two-dimensional code or barcode, or using NFC technology to access the database for query. Near-field communication (NFC) tags are actually a further extension of RFID, which is able to share information at close range wirelessly [146]. NFC tags include chips, antennas, and other devices, which have been greatly integrated into mobile devices such as smartphones, tablets, and laptops. By using a smart phone to make contact with the NFC tags, various information such as product composition, product date, and origin can be obtained to meet the basic needs of consumers [147,148]. NFC has the advantages of short-distance, high-frequency, and non-contact operation, and automatic identification.

Chen et al. [149] demonstrates that the use of NFC in agriculture-related supply chains will be crucial to their intelligent real-time management and traceability. Conti et al. [134] proposed an NFC-smartphone approach to food traceability for use in the corporate virgin olive oil supply chain. It stores the information in NFC tags, and the operator can access the product information from a smartphone. The system enables complete historical tracking of olive oil, allowing the consumer to obtain information such as production time, harvest in different fields/dates, grinding degree, etc. Mainetti et al. [135] created a gapless traceability system for the fresh vegetable supply chain using a combination of NFC, RFID, and data matrix technologies. Smart phones with NFC technology were applied to track, capture, and transmit management data in vegetable storage greenhouses. The results showed that the combination of the three technologies avoided the problems of high cost and information omission, improved the efficiency of traceability in the whole supply chain process, and was a reliable and efficient system. Ma et al. [136] studied the traceability system for cherries. This work marked the crucial information about cherries from manufacturing to processing, packaging, and sales, stored the food source, production time, and production batch in NFC chips, collected the information using smartphones, and enhanced the transparency of the entire cherry production chain.

The traceability system based on a smart phone and NFC label can help consumers to know various information about products and help managers to check and record products more effectively. Through the traceability system, suppliers can recall products with quality problems, thus guaranteeing product quality.

### 6.3. Other Food Traceability Equipment

One of the traceability systems, quick response (QR) codes, are frequently used on food packaging. By scanning QR codes with smartphones and other mobile devices, one can receive product information and product traceability [150]. Qian et al. [151] adopted the response surface optimization method to optimize the information readability of two-dimensional codes and improved the traceability performance of mobile phones. Yang et al. [152] built a new traceability code with origin location and encrypted authentication on a mobile platform and applied it to a real-time agricultural product certification and supervision system. This system solved the problem of counterfeiting and abuse of labels and improved the reliability of food traceability.

According to previous studies, stable isotope analysis is an effective method for determining the country of origin of fresh commodities like agricultural goods. The fundamental idea is that during the growing process, the naturally occurring stable isotopes in the food components are strongly linked to the natural systems (such as temperature, solar intensity, rainfall), soil background, and farming methods (fertilizer type, irrigation) [153]. Under different ecological soil environment and tillage methods, the stable isotope characteristics of crops have regional differences. Therefore, the origin of food can be traced by stable isotope analysis. At present, this technology is often used in conjunction with other analytical methods, and has achieved varying degrees of success in identifying and tracking vegetables, meat, seafood, and other foods. Song et al. [137] used stable isotope ratios (δ13C, δ15N, δ2H, δ18O, δ34S) to identify the connection between geographical differences and nutritional ingredient changes in Lentinus edodes in three different regions of China, so as to identify the origin of Lentinus edodes and realize classification. Pianezze et al. [138] performed the geographical identification and tracking of mutton based on stable isotope ratio analysis by focusing on the isotopic ratios of carbon, nitrogen, sulfur, hydrogen, and oxygen in different substrates, resulting in an accuracy of more than 90%.

In addition, chemometrics combined with near-infrared spectroscopy and chemometrics combined with nuclear magnetic resonance are also used in food traceability. Liu et al. [154] analyzed and predicted the chemical composition of tilapia fillets in several regions of China by using near-infrared reflectance spectroscopy. At the same time, combining with analog soft independent modeling (SIMCA), a tilapia origin tracing model was constructed, and tilapia fillets were correctly classified according to their origin. The results showed that near-infrared spectroscopy was an effective tool to trace the origin of tilapia fillets. Qian et al. [139] established a dual origin–variety tracing system by applying Fourier transform near-infrared spectroscopy and the partial least squares method, and identified and traced the origin and varieties of Baha Siber mung bean, realizing a new idea of brand protection. Schievano et al. [140] tracked the authenticity of acacia honey using MRI equipment to detect the sugar content of honey products. Huo et al. [155] used NMR-based metabolomics combined with PCA and DA to tell apart rice from different origins in China, which provided a valuable tool for identifying the provenance of the rice. 

Blockchain technology is also one of the technologies commonly used in food supply chain traceability systems. The researchers propose using blockchain technology to store food traceability information [156], improve the transparency and operational efficiency of the supply chain, and increase consumer trust in the retail food industry. Lei et al. [157] developed a trusted audit chain for blockchain agricultural traceability. Using this trusted blockchain-based audit chain not only increased the credibility of the traceable results, but also protected food safety and consumer privacy data. Blockchain technology has improved agricultural food traceability, according to Feng et al. [158], who reviewed the topic and came to the conclusion that this technology has some advantages in the field of food traceability and that further research into its use in sustainable food traceability systems should be conducted.

## 7. Conclusions and Future Outlook

This paper reviews developments in diverse intelligent testing equipment used for fresh food quality assessment and traceability. The application of such equipment for quality detection and traceability of fresh food in the supply chain involving harvesting, transportation and storage is summarized. Compared with traditional equipment, these devices have many advantages based on intelligent detection technology and advanced algorithms. They can detect and monitor the quality deterioration of fresh food online and non-invasively. At the same time, through the traceability system, one can obtain reliable information about the product source, variety, production date, and other information which can help ensure better quality of fresh food. However, the current intelligent detection equipment also has some limitations. For example, computer vision systems and the electronic nose, as well as other equipment, are easily affected by environmental conditions, so detection results can be unreliable. Hyperspectral imaging equipment and nuclear magnetic resonance equipment are both expensive and inconvenient to use. RFID tags bring a high cost and present recovery problems. Therefore, it is necessary to continuously enhance the portability and stability of the equipment, realize more efficient and reliable real-time detection of fresh food quality and ensure product quality at the consumer terminal.

## Figures and Tables

**Table 1:** Application of intelligent detection equipment for monitoring fresh food.

Equipment	Methods/Composition	Products	Function	Ref.
CV	The double-layer classifier detects ripeness and brown spots areas separately	Banana	Maturity detection classification	[19]
	3D stereo depth camera combined with deep learning algorithms	Potato	Evaluation of 3D shape defect detection	[20]
	Image segmentation techniques and vision system algorithms	Fig	Online quality classification	[21]
Intelligent packaging film	Sugarcane wax, butterfly pea flower extract, and agar as the substrate	Shrimp	Freshness detection	[22]
	Blueberry anthocyanins were encapsulated into protein–polysaccharide complexes	Mushroom	Storage freshness detection	[23]
	Time–temperature indicator (TTI) based on tyrosinase and tyrosine reactions	Turbot sashimi	Metamorphism detection	[24]
Electronic nose	The sensor array consists of six chemo-resistive sensors capable of detecting various volatile organic compounds	Broccoli	Freshness detection	[25]
	It consists of four specific metal oxide sensors, a photoionization detector, and a battery	Fish	Distinguishing fish	[26]
	Combined with near-infrared spectroscopy (NIR)	Pitaya	Predicting shelf life	[27]
	Combined with smart refrigerator and smart phone	Beef	Monitoring metamorphism	[28]
NIR	Handheld instrument: consists of a portable battery, built-in sensor, and results display	Pear	Checking the sugar content	[29]
	The measurement was made directly on the carcass surface using a fiber optic probe	Beef	Online inspection of beef quality	[30]
	It is composed of NIR testing, transportation part, classification operation and calculation control	Apple	Determination of soluble solid content	[31]
	It consists of nine halogen lamps and two spectral sensors	Pineapple	Testing the transparency of pineapple pulp	[32]
HSI	Light source: twenty tungsten halide spotlights; image acquisition: spectroscope, two different wavelength bandpass filters and monochrome camera	Citrus	Determination of citrus canker	[33]
	Using SpecimIQ hyperspectral camera combined with artificial neural network (ANN) algorithm	Pork	Quality prediction and freshness	[34]
	It consists of hyperspectral imager, halogen lamp, sample tray and computer, combined with minimum noise analysis method	Persimmon	Identification of health/injury	[35]
	Hyperspectral imager, light source and computer were used and then the algorithm is combined to build the model	Sweet potato	Defect detection	[36]
NMR	Combined with microwave vacuum drying (MVD)	Corn kernels	Real-time determination of moisture status during drying	[37]
	Lightweight handheld MR Scanner: a planar RF coil and a single-sided magnetic circuit form the sensor	Tuna and Beef	Quantitative analysis of fat content	[38,39]
	Transverse relaxation and magnetic resonance imaging were measured	Shrimp	Testing of dryness and quality	[40]
	Combined with deep learning neural network (DLNN) algorithm, lateral relaxation time was measured	Longan	Distinguishing between healthy fruit and defective fruit	[41]
US	It is composed of ultrasonic pulse generator, special ultrasonic transmitting and receiving transducer, digital oscilloscope, and computer	Apple	Evaluating maturity and shelf life	[42]
	The wave amplitude and propagation time at different distances on the pericarp are measured using ultrasonic probes	Avocado	Monitoring of internal changes	[43]
	The ultrasonic characteristics were measured by the through transmission mode, and then combined with the SVM method	Orange	Online grading	[44]
	It is composed of narrow-band ultrasonic transducer, ultrasonic pulse generator, digital oscilloscope, and computer	Raw ham	Determination of fat content	[45]

CV: computer vision; NIR: near-infrared spectrum; HSI: hyperspectral imaging; NMR: nuclear magnetic resonance; US: ultrasound.

**Table 2:** Application of intelligent traceability equipment for monitoring fresh food.

Equipment	Methods/Composition	Products	Function	Ref.
RFID	Barcode–RFID bidirectional conversion device	Beef	Segmentation of beef	[130]
	It is composed of RFID technology and information processing system	Pumpkin	Cold chain monitoring	[131]
	Consists of smart tags and commercial readers	Fish	Real-time traceability and cold chain monitoring	[132]
	RFID technology is combined with molecular analysis algorithms (DNAs)	Pork	Origin tracing and disease surveillance	[133]
NFC and Smartphones	The information is stored in NFC tags and accessed using smartphones	Olive oil	Complete history tracing	[134]
	The combination of NFC, RFID, and data matrix technology	Vegetables	Tracking and transmitting data in storage	[135]
	The information is stored in an NFC chip and accessed by a smartphone	Cherry	Improving information transparency	[136]
Stable isotope analysis	δ13C, δ15N, δ2H, δ18O, δ34S	Lentinus edodes	Identifying the origin	[137]
	13C,12C; 15N, 14N; 36S, 34S, 33S, 32S; 18O, 17O, 16O; 1H, 2H	Mutton	Geographic identification and tracing	[138]
NIR	NIR combined partial least squares	Mung bean	Origin and brand protection	[139]
NMR	The sugar content was detected by NMR	Honey	Authenticity detection	[140]

RFID: radio-frequency identification; NFC: near-field communication; NIR: near-infrared spectrum; NMR: nuclear magnetic resonance.
